# Obstetric Violence Through the Perspectives of Women and Midwives in Hospital Birth Settings: A Qualitative Study

**DOI:** 10.3390/healthcare14162579

**Published:** 2026-08-17

**Authors:** Yeliz Yıldırım Varışoğlu, Nazmiye Gürz, Meserret Aslan

**Affiliations:** 1 Department of Women’s Health and Diseases Nursing, Istanbul University Faculty of Nursing, Fatih, Istanbul 34381, Türkiye; yelizy@istanbul.edu.tr; 2 Department of Midwifery, Faculty of Health Sciences, Istanbul Atlas University, Kağıthane, Istanbul 34408, Türkiye; 242110010@st.atlas.edu.tr

**Keywords:** obstetric violence, respectful maternity care, traumatic childbirth, informed consent, midwives, qualitative research, hospital birth

## Abstract

**Background/Objectives:** Obstetric violence is increasingly recognised as a violation of women’s autonomy, dignity, and right to respectful maternity care, yet its interpretation may differ between women receiving care and midwives providing care within hospital systems. This study explored obstetric violence from the perspectives of women who perceived their childbirth as traumatic and midwives working in hospital birth settings. **Methods:** This qualitative study was conducted in one university hospital and two public hospitals in Türkiye. Data were collected through individual semi-structured interviews with 21 women who had experienced vaginal birth within the previous year and 20 midwives with labour and delivery experience. Interviews with women were conducted online, whereas interviews with midwives were conducted face-to-face. Interviews were audio-recorded, transcribed verbatim, and analysed using qualitative content analysis. Selected sociodemographic, obstetric, and organisational characteristics were collected descriptively to contextualise the findings. **Results:** Three interrelated themes were identified: lack of respectful maternity care, women’s emotional needs during childbirth, and systemic constraints affecting midwifery practice. Women described obstetric violence through emotional distress, loss of control, insufficient information, lack of consent, privacy violations, and imposed interventions. Midwives interpreted these experiences in relation to workload, institutional routines, physician-centred decision-making, and limited professional autonomy. **Conclusions:** Preventing obstetric violence requires improvements in communication, informed consent, privacy, emotional support, and system-level conditions that enable respectful, woman-centred care.

## 1. Introduction

Approximately 140 million births occur worldwide each year, and most are completed without major obstetric complications. However, favourable clinical outcomes do not necessarily ensure that childbirth is experienced as safe, respectful, or empowering by women. Increasing evidence indicates that insufficient communication, lack of informed consent, compromised privacy, and inadequate emotional support during labour and birth may lead women to perceive their childbirth experiences as traumatic [1,2,3]. Such experiences may extend beyond the intrapartum period, contributing to postpartum depression, symptoms of post-traumatic stress, difficulties in mother–infant bonding, and reduced trust in healthcare systems [3,4].

The concept of obstetric violence has evolved from an emphasis on overt physical abuse and non-consensual obstetric interventions to a broader understanding encompassing psychological, verbal, symbolic, relational, and structural forms of mistreatment during maternity care. The term gained formal legal recognition in Venezuela in 2007 and was initially used to describe the appropriation of women’s bodies and reproductive processes by healthcare professionals, particularly through dehumanised care, excessive medicalisation, and restrictions on women’s autonomy and decision-making rights [5,6]. Subsequent scholarship has expanded the concept beyond intentional acts committed by individual professionals to include routine practices, institutional cultures, professional hierarchies, and organisational conditions that may undermine women’s dignity, autonomy, and right to respectful maternity care. Mendes et al. [7] conceptualised obstetric violence as a multifactorial phenomenon shaped by the interconnected perspectives and practices of three central actors: parturient women, healthcare professionals, and healthcare institutions and services. From this perspective, women experience and interpret the interpersonal and bodily consequences of care, healthcare professionals influence how practices are communicated and implemented, and institutions shape these encounters through policies, resource allocation, routines, infrastructure, and hierarchical decision-making. Obstetric violence should therefore be understood not solely as deliberate individual misconduct, but as a relational and structural phenomenon emerging from the interaction between professional behaviours, institutional conditions, gendered power relations, and the organisation of maternity care.

This study was guided by the conceptual correlation framework proposed by Mendes et al. [7], which situates obstetric violence at the intersection of three interrelated actors: parturient women, healthcare professionals, and healthcare institutions and services. Within this framework, women’s narratives represent the experiential and relational consequences of maternity care; healthcare professionals’ accounts reflect how care practices are interpreted, negotiated, and implemented; and institutional conditions shape these encounters through organisational routines, professional hierarchies, staffing arrangements, resource limitations, and models of clinical decision-making. The framework was used as a sensitising conceptual lens rather than as a predetermined coding structure. It informed the inclusion of both women and midwives, the dimensions explored in the interview guides, and the comparative interpretation of interpersonal and structural aspects of obstetric violence, while allowing themes and subthemes to emerge inductively from participants’ narratives.

Empirical evidence suggests that obstetric violence most commonly manifests through failures in informed consent, breaches of privacy, unnecessary or routine medicalised interventions, harsh communication, and insufficient emotional support during childbirth [8,9]. Beyond caesarean section rates, national evidence indicates that obstetric violence is common in Türkiye. In a cross-sectional study involving 513 women who had given birth within the previous two years, Aşcı and Demirgöz Bal reported that 76.4% had experienced at least one form of obstetric violence. The most frequently reported forms were physical abuse and abandonment of care, each reported by 44.4% of participants, followed by non-consented care (26.5%) and non-dignified care (25.1%) [10]. These experiences may lead women to feel a loss of control and to perceive themselves as passive recipients rather than active participants in the birth process [11].

Recent evidence indicates that practices inconsistent with World Health Organization (WHO) recommendations recommendations continue despite increasing legal and professional attention to respectful maternity care. In a cross-sectional study involving 615 Portuguese women, Tavares et al. [12] found that many participants were not offered a choice of birth position, some were required to give birth in the lithotomy position, and women’s preferences and birth plans were frequently not respected. The study also identified continuing concerns related to informed consent, information provision, mobility, companionship, and women’s participation in decisions regarding their care. The authors emphasised the need for continuous training of healthcare professionals, stronger informed-consent procedures, and maternity care practices that respect women’s rights, preferences, and autonomy. These findings demonstrate that formal legal and professional advances alone may be insufficient to eliminate routine practices that women experience as coercive, disrespectful, or disempowering.

Obstetric violence also has important implications for healthcare professionals. Midwives, as primary caregivers during labour and birth, frequently witness women’s experiences of distress and disrespect. However, high workload, staffing shortages, time constraints, limited infrastructure, and physician-centred care models may restrict midwives’ ability to provide consistent woman-centred care [13,14]. These conditions may limit professional autonomy and create ethical tensions between midwives’ professional values and clinical realities [15]. The terminology surrounding obstetric violence remains debated internationally. Some scholars caution that the term “violence” may polarise professional dialogue or obscure the complexity of clinical decision-making, whereas others consider it necessary for making gendered, relational, and structural harms visible [6,13].

The Turkish context provides a relevant setting for examining obstetric violence, given the high level of medicalised childbirth and the ongoing need to strengthen respectful maternity care. National data indicate that caesarean section rates rose from 58.4% in 2021 to 60.1% in 2022 [16,17]. These findings indicate that the Turkish context cannot be understood solely through mode-of-birth statistics and should also be considered in relation to interpersonal conduct, consent practices, continuity of support, and organisational conditions. However, medicalisation is not limited to caesarean birth; it may also shape vaginal birth through routine interventions, limited informed consent, restricted mobility, insufficient privacy, and standardised institutional practices. Therefore, examining women’s and midwives’ perspectives on obstetric violence in hospital birth settings is important for understanding how respectful and woman-centred care can be strengthened in Türkiye. Traumatic childbirth and birth satisfaction are shaped not only by clinical outcomes but also by women’s perceptions of communication, control, emotional support, and participation in decision-making [3,4,12]. Accordingly, the implementation of woman-centred and respectful maternity care requires not only the prevention of overt mistreatment but also individualised information, meaningful consent, emotional support, and respect for women’s preferences. Midwife-led continuity-of-care models may further promote relational continuity, trust, and women’s participation throughout pregnancy and childbirth [18].

Although previous studies have documented women’s experiences of obstetric violence, the literature has predominantly examined the perspectives of women or healthcare professionals separately. Consequently, limited evidence is available on how the same maternity care practices are experienced by women and interpreted by midwives working within hospital systems, particularly in Türkiye. This gap restricts understanding of how interpersonal encounters, professional practices, and institutional conditions interact in the production or normalisation of obstetric violence. The present study therefore addressed the following research questions: (1) How do women who perceive their childbirth as traumatic describe experiences related to respectful and disrespectful maternity care? (2) How do midwives interpret these experiences and the professional and organisational conditions surrounding them? (3) In what ways do women’s and midwives’ perspectives converge or diverge regarding the interpersonal and structural dimensions of obstetric violence? Accordingly, this study aimed to explore and comparatively interpret obstetric violence from the perspectives of women with self-perceived traumatic childbirth experiences and midwives working in hospital birth settings. The innovative contribution of the study lies in bringing together care recipients’ lived experiences and care providers’ professional accounts within the same analysis, thereby revealing how practices experienced by women as coercive, distressing, or disrespectful may be understood by midwives in relation to institutional routines, professional hierarchies, workload, and restricted autonomy. This comparative perspective may contribute to the development of context-sensitive, respectful, trauma-informed, and woman-centred maternity care interventions in Türkiye.

## 2. Materials and Methods

### 2.1. Study Design

This study employed a qualitative descriptive design informed by a descriptive phenomenological orientation. This orientation was selected to privilege participants’ first-person accounts of their lived childbirth and professional experiences and to remain close to the meanings expressed in their narratives. It influenced data collection through the use of open-ended, non-leading questions that encouraged participants to provide detailed descriptions of concrete experiences rather than predefined evaluations. The study did not apply a formal phenomenological analytic method, such as Giorgi’s method, Colaizzi’s method, or interpretative phenomenological analysis. Instead, the interview data were analysed inductively using qualitative content analysis following Graneheim and Lundman’ approach. This approach enabled the systematic identification and interpretation of manifest and latent meanings while preserving the study’s focus on participants’ lived experiences.

The study was conceptually informed by Mendes et al.’s framework, which approaches obstetric violence through the interrelated perspectives of parturient women, healthcare professionals, and healthcare institutions and services. This framework was used as a sensitising lens to support the interpretation of the interpersonal, professional, and institutional dimensions reflected in participants’ narratives, rather than as a predetermined coding framework.

### 2.2. Study Setting

The study was conducted in Istanbul, Türkiye, in one university hospital and two public hospitals providing secondary- and tertiary-level maternity care. All three centres were urban hospital settings serving women from diverse socioeconomic and cultural backgrounds and providing antenatal, intrapartum, emergency obstetric, and postpartum services. Maternity care was delivered through multidisciplinary teams including obstetricians, midwives, nurses, and other healthcare personnel. The centres differed in their institutional structure, patient volume, and organisation of labour and delivery services. They were selected to capture variation between university and public hospital contexts and to support the interpretation of how organisational conditions may shape women’s and midwives’ experiences.

### 2.3. Participants and Sampling

Different sampling strategies were used for the two participant groups. Snowball sampling was employed to recruit women with self-perceived traumatic childbirth experiences, whereas purposive sampling was used to recruit midwives working in labour and delivery units.

Women were eligible if they were 18 years of age or older, had experienced a vaginal birth within the previous year in one of the participating hospitals, reported having a negative or traumatic childbirth experience, and were willing to participate in an in-depth interview.

Consistent with the phenomenological orientation of the study, traumatic childbirth was defined according to the woman’s own subjective perception rather than a clinical diagnosis or standardized assessment tool.

Women were recruited using a snowball sampling strategy. Initial participants were identified through the researchers’ professional networks, and subsequent participants were referred by women who had already been contacted. Potential participants were contacted by the research team via telephone after childbirth. Recruitment did not take place during labour or immediately after birth. At the initial contact, eligibility was assessed by asking whether the woman personally regarded her childbirth experience as negative or traumatic, whether the birth had occurred vaginally in one of the participating hospitals within the previous year, and whether she was willing to discuss her experience in an individual interview. No diagnostic instrument or predetermined obstetric-violence checklist was used to determine eligibility; the woman’s subjective appraisal of her childbirth experience was accepted. A total of 34 eligible women were approached. Twenty-one agreed to participate and completed the interviews. Thirteen declined because they did not wish to discuss their childbirth experiences or were unavailable during the data-collection period. No participant withdrew after providing consent or after the interview commenced.

Midwives were recruited using purposive sampling. Eligible participants were required to be currently working in a labour and delivery unit, to have professional experience in intrapartum care, and to be willing to participate in an individual interview. An invitation describing the purpose of the study, eligibility criteria, voluntary nature of participation, and confidentiality procedures was sent to the head midwife of each participating hospital. The head midwives distributed a Google Forms invitation link to all eligible midwives working in the labour and delivery units. Midwives who voluntarily expressed interest by completing the online form were subsequently contacted by the research team, provided with further information about the study, and invited to participate in an individual interview. A total of 20 midwives provided informed consent and completed the interviews. Because the invitation was distributed internally by the head midwives, the researchers did not have access to the total number of eligible midwives who received the invitation but did not express interest; therefore, a precise refusal rate could not be calculated. No midwife withdrew after providing consent or after the interview had commenced.

Recruitment continued until saturation was reached separately for women and midwives, as described in the Data Collection subsection.

### 2.4. Development of the Interview Guide

Semi-structured interview guides were developed separately for women and midwives to explore their experiences and perceptions of care during labour and childbirth. The guides were developed following a review of the literature on obstetric violence, mistreatment during childbirth, respectful maternity care, traumatic childbirth, and the organisational conditions shaping maternity care. More specifically, the questions were informed by Bohren et al.’s typology of mistreatment during facility-based childbirth [8]; the World Health Organization’s recommendations on respectful and woman-centred intrapartum care [18]; Mendes et al.’s conceptualisation of obstetric violence through the interconnected perspectives of parturient women, healthcare professionals, and healthcare institutions and services [7]; and Avcı and Kaydırak’s qualitative investigation of women’s experiences of obstetric violence in Türkiye [19].

These sources informed the principal dimensions explored in the interview guides, including women’s overall experiences of labour and childbirth, communication with healthcare professionals, information provision, informed consent, participation in decision-making, privacy and dignity, emotional support, mobility and birth preferences, routine obstetric interventions, disrespectful or demeaning interactions, and perceived neglect. The guide developed for midwives additionally explored professional autonomy, workload, staffing, institutional routines, interdisciplinary relationships, physician-centred decision-making, and organisational barriers to respectful maternity care. The guides were developed specifically for the present study and were not adapted from a previously validated interview instrument. The complete semi-structured interview guides used for women and midwives are provided in Supplementary File S1. Open-ended questions were used to encourage participants to describe their experiences in their own words without directing them towards predetermined responses. Questions concerning information, consent, decision-making, emotional support, and privacy were phrased as neutral invitations to describe how these processes occurred, rather than as yes/no questions that assumed either the presence or absence of mistreatment.

The guides followed a progressive structure that moved from broad, descriptive questions to more specific and potentially sensitive topics. The women’s guide comprised five sections: opening questions about the overall childbirth experience and pre-birth expectations; experiences of care during labour and birth; communication and emotional support; privacy and the birth environment; and retrospective reflections and recommendations. The midwives’ guide comprised six sections: professional background and the institutional birth environment; routine maternity care practices; respectful maternity care; perceptions and observations of obstetric violence; organisational and systemic factors; and recommendations for improving care. Both guides consisted primarily of open-ended core questions supported by optional follow-up probes. Descriptive questions were used to elicit participants’ experiences and perceptions, while probing questions invited clarification, elaboration, and concrete examples. The sequence was designed to establish rapport before addressing sensitive issues. In the interviews with women, the term “obstetric violence” was not introduced in the opening or core questions; participants were first invited to describe their labour and childbirth experiences in their own words. By contrast, midwives were asked directly about the meaning of obstetric violence only after discussing routine care and respectful maternity care, thereby placing the concept within their professional and organisational context.

The interview guides were reviewed by five academic experts who were independent of the data collection process. The expert group consisted of three specialists in women’s health and obstetric nursing and two specialists in midwifery. The experts had academic and research experience in childbirth care, qualitative research, respectful maternity care, and women’s health. They evaluated the interview guides in terms of clarity, relevance to the research aim, comprehensiveness, appropriateness of wording, cultural suitability for the Turkish context, question sequencing, and the potential for questions to be leading or judgemental. Based on their feedback, minor revisions were made to the wording, sequencing, and probing questions before data collection commenced. A formal pilot interview was not conducted before data collection. The clarity, relevance, comprehensiveness, cultural appropriateness, and non-leading formulation of the questions were instead evaluated through independent review by five academic experts. Although this expert-review process enabled revisions to the wording, sequencing, and probing questions, the absence of a separate pilot interview is acknowledged as a methodological limitation of the study. Women were recruited because they had self-identified their recent vaginal birth as a negative or traumatic experience. Consequently, interviews focused on participants’ narratives of labour and childbirth rather than asking whether they had experienced traumatic childbirth. Participants were first invited to describe their childbirth experiences in their own words, after which follow-up questions explored communication with healthcare professionals, informed consent, emotional support, and situations they perceived as respectful, disrespectful, supportive, or distressing.

No substantive changes were made to either interview guide after data collection commenced. The same core questions and sequence were used across all interviews to maintain consistency. However, consistent with the semi-structured design, the wording and order of follow-up probes were used flexibly according to the flow of each participant’s narrative. These adaptations were limited to clarification and elaboration and did not introduce new interview dimensions or alter the focus of the study.

### 2.5. Data Collection

Data were collected through individual semi-structured interviews conducted between March and September 2025. Separate interview guides were used for women and midwives. Before each interview, participants received verbal and written information regarding the purpose and procedures of the study, the voluntary nature of participation, confidentiality, and their right to decline any question or withdraw from the study at any time without consequence. Written informed consent was obtained from all participants before the interviews.

Interviews with women were conducted individually through a secure videoconferencing platform at a mutually convenient time. The online format was selected to facilitate participation and provide scheduling flexibility. Interviews with midwives were conducted face-to-face in a quiet, private room within their hospitals to protect confidentiality and minimise workplace interruptions. All interviews lasted approximately 30 min, with a range of 25–40 min.

In addition to the semi-structured interviews, brief structured contextual forms were administered separately to women and midwives. The form completed by women documented gestational age at birth and their self-reported experiences of selected intrapartum practices, including labour induction, enema administration, information provision, informed consent, physical violence, episiotomy, and post-birth skin-to-skin contact. The form completed by midwives documented their perceptions of routine maternity care practices and organisational conditions in their current labour and delivery units, including the availability of individual labour rooms, information provision, routine induction, enema administration, and continuous fetal monitoring. These forms were not observational checklists completed by the researchers and did not constitute independent verification of clinical practices. The descriptive data were collected solely to contextualise the qualitative narratives and were not used for inferential analysis or direct statistical comparison between women and midwives. Because the number of participants from each centre was limited and centre-specific reporting could increase the risk of institutional or participant identification, the structured data were aggregated across the participating hospitals, and no centre-level comparison was conducted.

With participants’ permission, the online interviews with women were audio-recorded using the recording function of a secure videoconferencing platform, whereas the face-to-face interviews with midwives were recorded using a password-protected digital audio recorder. Following each interview, the recordings were transferred to an encrypted, access-restricted storage system and subsequently transcribed verbatim. During and immediately after each interview, the interviewer documented field notes concerning the interview context, relevant non-verbal expressions observed during the face-to-face interviews, and preliminary analytical reflections. These field notes supported the interpretation of the interview data and informed the concurrent analytical process.

Several strategies were used to minimise social desirability bias. Participants were informed that there were no right or wrong answers and that the researchers were interested in their personal experiences and perspectives rather than socially or professionally desirable responses. They were encouraged to discuss both positive and negative experiences openly. All interviews were conducted individually, and participants were assured that their responses would remain confidential and that identifying information would not be included in the transcripts or study reports. The interviewers adopted a neutral and non-judgemental approach and used open-ended questions and non-directive follow-up probes. Women were assured that their responses would not affect the healthcare services they received, whereas midwives were informed that their responses would not affect their employment or professional responsibilities.

Data collection and preliminary analysis were conducted concurrently. Saturation was assessed separately for the women’s and midwives’ datasets by the two researchers involved in data collection and analysis (N.G. and Y.Y.V.). Following each interview, the researchers reviewed the transcript and field notes and compared the emerging codes and categories with the existing analytical framework. They then discussed whether the interview contributed a substantively new code, category, perspective, or interpretive dimension relevant to the research questions. Saturation was considered to have been reached when successive interviews generated only repetition or further elaboration of previously identified content and no new analytically meaningful codes or categories emerged. The decision to conclude recruitment was made jointly during researcher consensus discussions rather than by a single researcher. The final interviews conducted within each participant group were used to confirm the stability and comprehensiveness of the emerging thematic structure.

### 2.6. Researcher Characteristics and Reflexivity

Data collection was conducted by two female researchers, N.G. and Y.Y.V. N.G. is a specialist midwife working in clinical practice, with professional experience in maternity care and an academic background in midwifery. Y.Y.V. is an associate professor specialising in women’s health and obstetric nursing, with academic and research experience in maternity care and qualitative research. Both researchers were familiar with childbirth care and the principles of respectful maternity care. Before each interview, participants were informed about the researchers’ professional backgrounds and the purpose of the study. Neither interviewer had previously provided clinical care to the participating women or held a supervisory, managerial, or employment-related relationship with the participating midwives.

Before data collection, N.G. and Y.Y.V. discussed the study objectives, interview procedures, and the potential influence of their clinical and academic backgrounds on the research process. Reflexive field notes were maintained throughout data collection to document contextual observations, interview impressions, and preliminary interpretations. The researchers used open-ended, neutral, and non-judgemental questions and avoided expressing personal opinions or clinical evaluations during the interviews. Regular reflexive discussions were held throughout data collection and analysis to examine how their professional experiences and prior knowledge might influence questioning, coding, and interpretation. Coding and analytical decisions were reviewed collaboratively, and disagreements were resolved through discussion and consensus.

### 2.7. Data Analysis

Interview data were analysed inductively using qualitative content analysis as described by Graneheim and Lundman. No pre-existing coding framework, predetermined categories, or themes were imposed on the data. Instead, codes, subcategories, categories, and overarching themes were developed from participants’ narratives through an iterative analytical process. Audio recordings were transcribed verbatim and checked against the original recordings to ensure accuracy. The transcripts were read repeatedly by N.G. and Y.Y.V. to achieve immersion in the data and obtain a comprehensive understanding of participants’ experiences. Meaning units relevant to the research aim were identified, condensed, and assigned initial codes. Coding was performed independently by N.G. and Y.Y.V. The independently developed coding frameworks were subsequently compared during a series of researcher meetings. Differences in coding, categorisation, and interpretation were discussed in relation to the original transcripts and resolved through consensus. Similar codes were grouped into subcategories and categories, from which the overarching themes were developed.

MAXQDA (2024) software was used to facilitate data management, coding, retrieval of quotations, and organisation of categories and themes. Throughout the analytical process, constant comparison was employed within and across the interviews of women and midwives to identify similarities, differences, and recurring patterns in their experiences.

Following completion of the qualitative analysis, an author-developed two-case model was created using MAXMaps in MAXQDA 2024 (VERBI Software, Berlin, Germany) to visually synthesise the final themes and comparative patterns identified in women’s and midwives’ accounts. The model illustrates group-specific and shared interpretive dimensions derived from the final thematic framework. It was used as a qualitative visual synthesis and does not represent coding frequencies, prevalence estimates, or statistical comparisons.

### 2.8. Trustworthiness

The trustworthiness of the study was established according to the criteria of credibility, dependability, confirmability, and transferability proposed by Lincoln and Guba.

Credibility was enhanced through investigator triangulation, independent coding by two researchers, consensus meetings, and continuous comparison throughout the analysis. Dependability was supported by maintaining a transparent description of the research procedures, including participant recruitment, data collection, and analytical decisions. Formal member checking or return of the preliminary findings to participants was not conducted. Credibility was instead strengthened through independent coding, investigator triangulation, regular analytical discussions, consensus-based resolution of disagreements, constant comparison within and across participant groups, and the inclusion of direct participant quotations.

Confirmability was strengthened through independent coding and collaborative discussions between the researchers to minimise individual interpretative bias and ensure that the findings reflected participants’ accounts rather than researchers’ preconceptions.

Transferability was promoted by providing detailed descriptions of the study setting, participant characteristics, sampling procedures, and analytical process, enabling readers to determine the applicability of the findings to similar contexts.

### 2.9. Ethical Considerations

Ethical approval for this study was obtained from the Istanbul Atlas University Non-Interventional Ethics Committee (Date: 17 February 2025; Decision No: 02/27). The study was conducted in accordance with the principles of the Declaration of Helsinki. Written and verbal informed consent was obtained from all participants before data collection. Participants were informed about the purpose of the study, the voluntary nature of participation, confidentiality of data, and their right to withdraw from the study at any time without providing a reason.

Because the study involved women who described their childbirth experiences as traumatic, particular attention was paid to participants’ emotional comfort during the interviews. Participants were informed that they could decline to answer any question, pause the interview, or terminate the interview at any stage. Interviews were conducted in environments that ensured participants’ privacy and confidentiality. If emotional discomfort occurred during the interview, the interview was paused or discontinued according to the participant’s preference.

All audio recordings and transcripts were anonymised, and participants were coded as P1–P21 for women and M1–M20 for midwives. The data were stored securely and used solely for research purposes.

The study is reported in accordance with the Consolidated Criteria for Reporting Qualitative Research (COREQ) guideline.

## 3. Results

### 3.1. Participant Characteristics

A total of 41 participants were included in the study, comprising 21 women who reported a recent traumatic childbirth experience and 20 midwives working in labour and delivery units. The women were between 23 and 45 years of age, with a mean age of 30.4 ± 7.4 years. Most women were married (90.5%), lived in nuclear families (81.0%), and had completed high school or university education (95.2%). Fourteen women (66.7%) were employed as civil servants or were self-employed, and 90.5% had children before the index birth. The participating midwives had a mean age of 33.5 ± 11.7 years, a mean of 11.2 ± 11.9 years of total professional experience, and a mean of 9.1 ± 8.6 years of labour and delivery unit experience. Only these three characteristics were collected from the midwives. Participant characteristics are presented in aggregate form in Table 1.

### 3.2. Women’s Childbirth-Related Experiences

Women’s childbirth-related experiences are presented in Table 2. The mean gestational age at birth was 38.24 ± 1.95 weeks (range: 34–41 weeks). Eight women (38.1%) reported receiving labour induction, whereas six (28.6%) underwent enema administration during labour. Nine participants (42.9%) reported that informed consent had not been obtained before clinical procedures, while six women (28.6%) stated that they had not received sufficient information during labour. Episiotomy was performed in 52.4% of participants, and skin-to-skin contact following birth was reported by 66.7% of women. Only two participants (9.5%) reported experiencing physical violence during childbirth.

### 3.3. Midwives’ Reports on Routine Maternity Care Practices

Midwives’ reports regarding routine maternity care practices and organisational characteristics are summarised in Table 3. Most participants reported that women were routinely informed about labour procedures (70.0%), while 60.0% indicated that individual labour rooms were available. Continuous fetal monitoring was reported by 65.0% of midwives, whereas routine labour induction and enema administration were reported less frequently (10.0% and 40.0%, respectively). These findings provide contextual information regarding routine maternity care practices in the participating hospitals and serve as background for interpreting the qualitative findings.

### 3.4. Qualitative Findings

Qualitative content analysis generated three overarching themes describing women’s and midwives’ perspectives on obstetric violence: (1) Lack of Respectful Maternity Care, (2) Women’s Emotional Needs During Childbirth, and (3) Systemic Constraints Affecting Midwifery Practice. Although both participant groups frequently referred to similar childbirth practices, they interpreted these experiences differently. Women primarily described the emotional, psychological, and embodied consequences of care practices, whereas midwives interpreted the same situations within organisational, professional, and structural constraints. The themes, subthemes, representative quotations, and comparative interpretations are presented in Table 4.

#### 3.4.1. Lack of Respectful Maternity Care

Lack of respectful maternity care emerged as the dominant theme across both participant groups. Women consistently described respectful maternity care as extending beyond the absence of physical harm. For them, respectful care meant receiving clear information, participating in decisions concerning their own bodies, having their privacy protected, and being treated with dignity throughout labour and birth. Midwives similarly acknowledged these principles as fundamental to quality maternity care; however, they frequently explained shortcomings in respectful care in relation to organisational routines, physician-centred decision-making, and the realities of hospital practice. Three closely related subthemes were identified: (i) inadequate information and compromised informed consent, (ii) compromised privacy and dignity, and (iii) interventions perceived as imposed.

##### Inadequate Information and Compromised Informed Consent

Women repeatedly described inadequate information as one of the most distressing aspects of their childbirth experience. Although some participants reported receiving brief explanations before procedures, these explanations were often perceived as insufficient and did not allow meaningful participation in decision-making. Rather than experiencing informed consent as a collaborative process, many women felt that decisions had already been made before they were informed.

One participant explained:

*“No, I was not given enough information, and my consent was not obtained. It was more like they told me and then did it. There were times when I did not receive answers to all my questions. An enema was performed, but nobody asked whether I agreed. I think it was simply considered a routine procedure.”*

(P5)

Beyond procedural consent, women also described informational uncertainty regarding the condition of their babies and the progress of labour. These narratives suggest that the need for information extended beyond clinical interventions and reflected a broader desire to remain actively involved in childbirth.

Several women stated that they did not object to clinical interventions themselves but wished to understand why they were necessary and to be involved in the decision-making process. The absence of adequate explanations reduced their sense of control and left them feeling excluded from their own childbirth experience.

Midwives similarly acknowledged that informed consent was not always achieved in routine clinical practice. However, unlike women, they frequently attributed these shortcomings to physician-led decision-making and established institutional routines rather than to intentional neglect.

*“The woman’s consent is not always obtained. Induction is used in high-risk pregnancies, but unfortunately it is sometimes performed even when no clear risk is present.”*

(M8)

*“For many physicians, enema has become a routine practice. Most women initially do not want the procedure, but after the physician explains it, they usually agree.”*

(M11)

Together, these findings suggest that women interpreted insufficient information and limited informed consent as a loss of autonomy and participation, whereas midwives understood these shortcomings largely within the context of routine clinical practice and physician-led care.

##### Compromised Privacy and Dignity

Maintaining privacy during labour and birth emerged as another fundamental component of respectful maternity care. Women described feeling uncomfortable when multiple healthcare professionals entered the delivery room, when unnecessary personnel were present during examinations, or when their bodily privacy was inadequately protected. These experiences contributed to feelings of vulnerability, embarrassment, and loss of dignity.

One participant stated:

*“There were so many people around me while I was giving birth that I felt as though I were an object on display.”*

(P8)

Some women reported that the continuous presence of different healthcare professionals during labour made them feel observed rather than cared for, reinforcing feelings of exposure and vulnerability.

Midwives also recognised privacy as an area requiring improvement. Although they acknowledged that maternity services had become more sensitive to privacy over time, they described persistent organisational barriers, including frequent movement of staff and inadequate control of access to delivery rooms.

*“Compared with my first years in practice, there is greater attention to privacy now, but too many people still enter and leave the room. Physicians, midwives, nurses, and support staff come in repeatedly, doors are often left open, and women become very uncomfortable.”*

(M8)

While women primarily described the emotional consequences of compromised privacy, midwives interpreted these experiences as consequences of organisational routines and the physical organisation of hospital birth settings.

##### Interventions Perceived as Imposed

Women also described several obstetric interventions as distressing when they were performed without adequate preparation or explanation. Importantly, women rarely questioned the clinical necessity of interventions themselves; rather, they questioned the absence of explanation, preparation, and involvement in decision-making. Instead, they emphasised that the manner in which procedures were introduced and implemented determined whether they were experienced as supportive or traumatic.

*“When I could not push correctly, they pressed on my abdomen. I know they did it for my baby, but if someone had explained how painful it would be, I could have prepared myself.”*

(P3)

Rather than rejecting obstetric interventions themselves, women questioned the absence of preparation and explanation before these procedures. Their accounts suggest that the experience of coercion emerged primarily from how interventions were introduced rather than from the interventions alone.

Another participant recalled:

*“When I asked whether my baby was well, they told me not to worry, but they did not place my baby in my arms.”*

(P11)

Midwives acknowledged that many interventions were implemented within physician-directed clinical protocols and recognised that communication surrounding these procedures was not always adequate. Their accounts suggested that routine interventions were frequently shaped by institutional expectations rather than by individualised woman-centred care.

##### Theme Synthesis

Both women and midwives agreed that respectful maternity care depends on effective communication, meaningful informed consent, protection of privacy, and women’s active participation in decisions regarding their care. However, the two groups interpreted failures in respectful care differently. Women described these experiences through their immediate emotional consequences, including uncertainty, vulnerability, loss of dignity, and diminished control. Midwives explained the same practices primarily through organisational routines, physician-centred decision-making, and institutional constraints. These contrasting interpretations demonstrate how the same clinical practices may be understood differently depending on whether they are experienced by women or enacted within organisational routines.

#### 3.4.2. Women’s Emotional Needs During Childbirth

Women’s emotional needs constituted the second overarching theme. Women consistently described childbirth as both a physical and an emotional experience characterised by fear, loneliness, helplessness, and a profound need for reassurance. Emotional support was viewed as an essential component of safe childbirth rather than an optional aspect of care. Midwives also recognised women’s emotional vulnerability but described considerable difficulty in providing continuous emotional support because of organisational constraints and competing clinical responsibilities.

##### Emotional Vulnerability During Childbirth

Many women described childbirth as an emotionally overwhelming experience in which they felt isolated and unsupported.

*“It was my first birth, and I was completely alone. I wish I could have given birth in my own country.”*

(P8)

Another participant stated:

*“I have never felt so alone in my life.”*

(P19)

Several women explained that emotional isolation was particularly intense because childbirth, which they had expected to be a supportive experience, became an experience of facing pain and uncertainty alone.

These narratives illustrate that emotional distress was closely linked to the absence of continuous support during labour.

##### Need for Reassurance and Compassionate Care

Women emphasised that empathy and encouragement from healthcare professionals could substantially influence their childbirth experience.

*“I told them I was in pain, but they simply said, ‘This is childbirth.’ The midwife examining me had long fingernails, which hurt me. When I told her, she ignored me completely.”*

(P11)

Another participant explained:

*“I didn’t really understand what was happening or what I was supposed to do. I lost my confidence. I felt exhausted, and instead of helping me, they scolded me, saying, ‘Don’t you even know how to push?’”*

(P7)

Women associated insensitive communication not only with emotional distress but also with diminished confidence in their own ability to give birth. Feeling criticised during labour intensified feelings of inadequacy and loss of control.

Rather than viewing these interactions as isolated incidents, women described them as experiences that intensified helplessness and emotional distress.

##### Midwives’ Perspectives

Midwives recognised emotional support as an essential component of respectful maternity care but explained that organisational demands frequently prevented them from remaining continuously present with labouring women.

*“Unfortunately, women are not allowed to eat. I know this is not how care should ideally be provided, but according to the physician’s instructions, women are kept fasting to minimise potential risks.”*

(M19)

*“Even when the NST is reactive, we sometimes have to continue monitoring because some physicians do not want to assume responsibility.”*

(M16)

Midwives’ narratives suggest that organisational priorities frequently displaced emotional care. Clinical tasks and institutional protocols often took precedence over opportunities to remain physically and emotionally present with labouring women.

##### Theme Synthesis

Women experienced the absence of emotional support as loneliness, fear, and abandonment, whereas midwives attributed these shortcomings primarily to workload, physician-directed clinical routines, and competing organisational demands. Together, these findings suggest that emotional support was not perceived by women as an additional component of maternity care but as an essential condition for feeling safe, respected, and involved during childbirth. Midwives recognised these needs but described organisational barriers that limited their capacity to provide continuous emotional support.

#### 3.4.3. Systemic Constraints Affecting Midwifery Practice

The third theme captured the organisational and structural conditions that shaped maternity care and influenced midwives’ ability to provide respectful, individualised, and woman-centred care. Three subthemes were identified: restricted professional autonomy, physician-dominated decision-making, and moral distress arising from organisational constraints.

##### Restricted Professional Autonomy

Midwives consistently described limited professional autonomy as one of the principal barriers to providing respectful maternity care.

*“Sometimes we know what would be best for the woman, but we cannot act independently.”*

(M3)

Another participant explained:

*“Because there are not enough staff, women’s mobility is often restricted. Unfortunately, this is rarely explained to the woman.”*

(M7)

Midwives repeatedly distinguished between the care they believed women should receive and the care they were practically able to provide. This discrepancy emerged as a recurring source of professional frustration.

Several midwives explained that staffing shortages, overcrowded delivery units, and increasing clinical demands limited their opportunities to provide continuous, individualised care. Consequently, professional ideals frequently conflicted with the realities of everyday practice.

##### Physician-Dominated Decision-Making

Participants repeatedly referred to physician-centred decision-making as a major organisational factor shaping childbirth care.

*“Even when the NST is reactive, continuous monitoring, routine enemas, and labour induction are sometimes maintained because some physicians do not want to assume responsibility.”*

(M16)

Defensive clinical practice was described as an important organisational influence on routine interventions. Midwives explained that decisions were frequently driven by concerns about professional responsibility rather than women’s individual preferences.

Women experienced these organisational realities through rushed and impersonal care.

*“Everything happened so quickly. I felt like I was part of a routine rather than an individual.”*

(P18)

Women rarely referred directly to organisational problems. Instead, they described the lived consequences of these structural conditions through rushed care, limited communication, and reduced involvement in decision-making.

##### Moral Distress

Midwives described experiencing tension when institutional expectations conflicted with their professional values.

*“We often know that women need more time, information, and support, but we are not always able to provide it because we must follow institutional routines.”*

(M14)

Women indirectly reflected these organisational constraints through feelings of exclusion from decision-making.

*“I felt as though everything was happening around me, but I was not part of the decisions.”*

(P16)

Although midwives acknowledged that some routine practices were inconsistent with respectful maternity care, they frequently described themselves as having limited authority to challenge physician-led decisions or established institutional routines. Consequently, they experienced tension between their professional values and the care they were able to provide.

##### Theme Synthesis

Women described the consequences of organisational constraints through feelings of loss of control, emotional distress, and insufficient individualised care. Midwives explained these same experiences through staffing shortages, hierarchical decision-making, institutional routines, and restricted professional autonomy. Taken together, these findings demonstrate that women and midwives occupied different positions within the same maternity care system. Women described the immediate consequences of organisational constraints through their lived experiences of childbirth, whereas midwives explained the structural mechanisms that shaped those experiences. These complementary perspectives illustrate that obstetric violence is experienced not only through interpersonal interactions but also through the organisational structures that shape everyday maternity care.

#### 3.4.4. Cross-Group Comparative Synthesis

A cross-group comparison revealed both convergence and divergence between women’s and midwives’ accounts. The two participant groups converged in recognising clear communication, meaningful informed consent, privacy, emotional support, and participation in decision-making as essential components of respectful maternity care. Midwives did not generally dispute the importance of these principles and, in several accounts, explicitly acknowledged that routine practice did not always reflect them.

The principal divergence concerned how shortcomings in care were interpreted. Women described inadequate information, limited participation, imposed interventions, and lack of emotional support through their immediate consequences, including fear, loneliness, exposure, loss of control, and diminished dignity. Midwives, by contrast, more frequently framed the same shortcomings in relation to workload, staffing limitations, physician-directed decisions, institutional routines, and restricted professional autonomy. Thus, women’s narratives emphasised the consequences of care, whereas midwives’ narratives emphasised the conditions under which care was delivered.

The comparison also revealed an important tension between recognition and responsibility. Midwives demonstrated awareness of the principles of respectful maternity care and sometimes identified practices that were inconsistent with these principles. However, they frequently positioned these practices within organisational constraints rather than describing the extent to which individual communication styles, attitudes, or discretionary actions might also shape women’s experiences. Organisational explanations therefore appeared to function both as descriptions of genuine structural barriers and, potentially, as ways of distancing individual professionals from responsibility for disrespectful care.

Women’s accounts further indicated that the experience of obstetric violence was not determined solely by whether an intervention occurred. Several women did not reject the possible clinical need for procedures; instead, they objected to inadequate explanation, lack of preparation, and exclusion from decision-making. This distinction suggests that the boundary between clinically necessary intervention and an experience perceived as coercive or traumatic may depend substantially on communication, consent, relational conduct, and the manner in which professional authority is exercised.

Taken together, the findings show that systemic constraints and interpersonal practices should not be treated as competing explanations. Organisational conditions may limit the time, autonomy, and resources available to midwives, while individual attitudes and communication behaviours may still influence whether women experience care as respectful or dehumanising. Obstetric violence therefore emerged as a relational phenomenon produced through the interaction of institutional structures, professional hierarchies, routine practices, and everyday interpersonal encounters.

**Figure 1:**
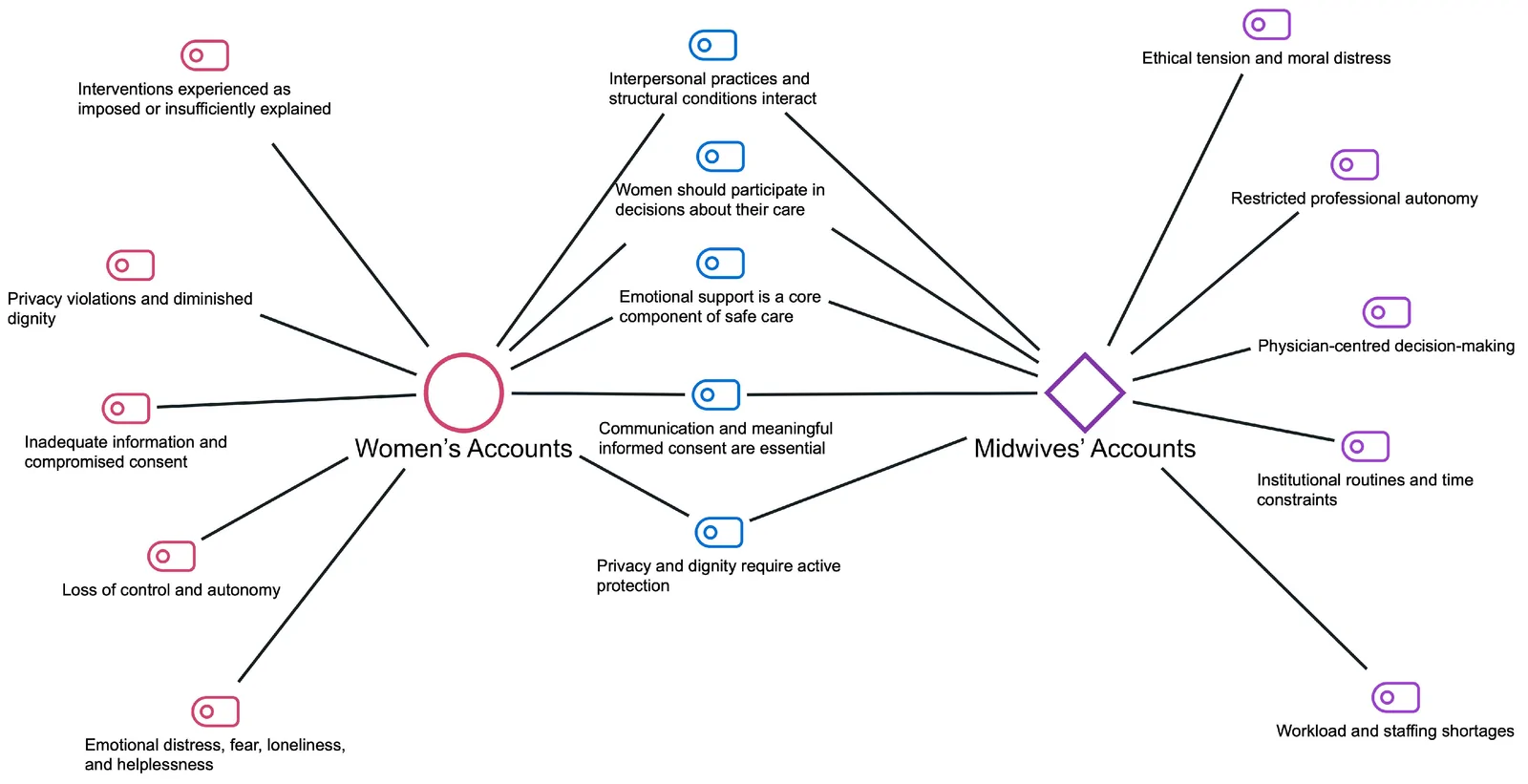
Two-case qualitative comparison of women’s and midwives’ perspectives. The MAXMaps model visually synthesises group-specific and shared dimensions identified in the final thematic analysis. Pink symbols represent dimensions emphasised in women’s accounts, purple symbols represent dimensions emphasised in midwives’ accounts, and blue symbols represent shared concerns. The model is an interpretive qualitative synthesis and does not display coding frequencies or statistical comparisons.

The two-case model provided a visual synthesis of the areas of convergence and divergence between women’s and midwives’ accounts (Figure 1). Women’s accounts primarily emphasised the emotional, relational, and autonomy-related consequences of maternity care, including fear, loneliness, loss of control, inadequate information, compromised consent, privacy violations, and interventions perceived as imposed. Midwives’ accounts more strongly emphasised workload, staffing shortages, institutional routines, physician-centred decision-making, restricted professional autonomy, and moral distress. Despite these differing interpretive positions, both groups identified communication, meaningful informed consent, privacy and dignity, emotional support, women’s participation in decision-making, and the interaction between interpersonal practices and structural conditions as central concerns.

## 4. Discussion

This study explored obstetric violence through the perspectives of women who perceived their childbirth as traumatic and midwives working in hospital birth settings. The findings showed that women mainly interpreted obstetric violence through emotional distress, loss of control, inadequate information, insufficient consent, privacy violations, and non-consensual or imposed interventions. In contrast, midwives tended to interpret similar experiences within the context of workload, institutional routines, physician-centred decision-making, limited professional autonomy, and organisational constraints. This divergence between women’s experiential accounts and midwives’ organisational explanations represents one of the main contributions of the study.

The findings support the growing body of evidence indicating that respectful maternity care is not limited to the absence of physical abuse, but also includes communication, dignity, informed choice, privacy, emotional support, and women’s active participation in decision-making [20,21]. In the present study, women’s accounts of feeling uninformed, invisible, exposed, or excluded from decisions reflect core dimensions of disrespectful and abusive maternity care described in previous international and national studies [2,22,23]. These findings suggest that obstetric violence should be understood not only through overt acts of mistreatment, but also through routine care practices that undermine women’s autonomy, dignity, and sense of control.

Lack of informed consent emerged as a central component of women’s traumatic childbirth narratives. Women did not describe consent merely as a procedural requirement, but as a matter of bodily autonomy, dignity, and participation in care. This finding is consistent with the recent literature emphasising that informed consent in maternity care should be understood as an ongoing communicative and ethical process rather than a signed form or brief explanation [24]. In this study, some women perceived procedures as imposed or performed without sufficient explanation, which points to a gap between clinical routines and woman-centred care. Such gaps may be particularly harmful during labour, when women are physically vulnerable, emotionally distressed, and highly dependent on healthcare professionals for information and reassurance.

An important nuance in the findings is that women did not consistently reject obstetric interventions or professional expertise. Several participants recognised that an intervention might have been clinically necessary or intended to protect the baby, yet still experienced the process as distressing because they were not adequately informed, prepared, or involved in decision-making. This distinction suggests that the experience of coercion cannot be inferred solely from whether an intervention was performed. Rather, it may emerge from the relational and communicative manner in which professional authority is exercised. A clinically indicated procedure may still be experienced as disrespectful when explanation, consent, emotional preparation, and opportunities for participation are absent. Conversely, clear communication and meaningful involvement may reduce feelings of coercion even when urgent intervention is required. A related tension was evident in midwives’ accounts. Midwives generally endorsed the principles of respectful maternity care and recognised that some routine practices were inconsistent with these principles. Nevertheless, they also described participating in or being unable to challenge practices they considered suboptimal. This tension between professional knowledge and everyday practice indicates that awareness of respectful care does not automatically translate into its consistent implementation. It also highlights the interaction between professional agency, institutional hierarchy, normalised routines, and moral distress.

Privacy was another important dimension of respectful maternity care. Women’s accounts of exposure and discomfort in crowded birth environments suggest that privacy violations may intensify feelings of vulnerability and loss of control. Similar findings have been reported in recent studies from Türkiye, where women described obstetric violence through lack of privacy, non-consensual procedures, loss of autonomy, and emotional harm [20,21]. In the present study, midwives’ reports also pointed to environmental and organisational limitations, indicating that privacy cannot be addressed only as an individual professional responsibility. Protecting privacy requires institutional arrangements, adequate physical infrastructure, and care environments that support dignity during labour and birth.

Women’s emotional needs during childbirth formed another important dimension of the findings. Participants described fear, loneliness, uncertainty, and the need for reassurance during labour. The absence of emotional support intensified their perception of childbirth as traumatic. This finding is consistent with recent evidence showing that disrespectful or inadequate treatment during childbirth may be associated with postpartum post-traumatic stress symptoms, whereas supportive communication, social support, and early skin-to-skin contact may function as protective factors [25,26]. In this context, emotional support should not be viewed as an optional or secondary aspect of maternity care, but as a central component of safe, respectful, and trauma-informed childbirth care.

Midwives recognised emotional support as an essential component of woman-centred care, but they also described difficulty providing continuous support because of high workload, staffing shortages, and competing clinical responsibilities. This finding demonstrates that emotional neglect during childbirth may not always result from individual indifference. Rather, it may reflect structural conditions that prevent midwives from practising in accordance with their professional values. Recent evidence on midwives’ perspectives similarly shows that professional hierarchies, institutional pressures, workload, and limited autonomy may place midwives in ethically difficult positions and restrict their ability to provide respectful care [27].

Systemic constraints affecting midwifery practice constituted a key finding of this study. Midwives described limited professional autonomy, physician-centred decision-making, staffing pressures, and routine institutional practices as barriers to respectful and woman-centred care. These findings are consistent with recent meta-synthesis evidence indicating that obstetric violence should be understood within a multidimensional framework, including individual, organisational, professional, and societal factors [27]. The ethical tension expressed by midwives in this study suggests that they may also experience moral distress when they recognise women’s needs but cannot respond adequately within existing institutional structures.

The comparison of women’s and midwives’ narratives is particularly important. Women experienced the consequences of systemic constraints as rushed, impersonal, and standardised care, whereas midwives identified the organisational mechanisms behind these experiences. This mismatch may contribute to the normalisation of practices that professionals perceive as routine, necessary, or unavoidable but women experience as disrespectful, coercive, or traumatic. Therefore, obstetric violence should not be reduced to isolated acts of individual misconduct. Instead, it should be understood as a relational and structural phenomenon embedded in the organisation of maternity care.

However, recognising the influence of structural conditions should not obscure the role of individual professional attitudes and discretionary behaviours. Workload, staffing shortages, institutional routines, and hierarchical decision-making may restrict the time and autonomy available to midwives, but they do not necessarily determine how every interaction with a woman unfolds. Even within similarly demanding clinical environments, professionals may differ in the extent to which they provide explanations, acknowledge women’s preferences, protect privacy, offer reassurance, and communicate respectfully. These differences may be shaped by professional values, communication skills, ethical sensitivity, previous training, personal beliefs regarding childbirth, and the degree to which routine practices are critically questioned. Systemic constraints and individual attitudes should therefore be understood as interacting rather than competing explanations: organisational conditions may increase the likelihood of impersonal or standardised care, while individual professional conduct may either reinforce or mitigate their effects on women’s experiences.

The findings also raise the question of why professionals working under comparable organisational pressures may differ in their ability to provide respectful care. Workload alone is unlikely to explain this variation. Individual and team-level protective factors may include stronger commitment to woman-centred care, greater ethical sensitivity, communication competence, reflective practice, supportive professional role models, previous training, and confidence in advocating for women within hierarchical clinical environments. Conversely, the normalisation of routine interventions, paternalistic beliefs, limited awareness of obstetric violence, emotional exhaustion, and workplace cultures that discourage questioning may increase the likelihood of dismissive or impersonal interactions. These factors were not directly compared among the participating midwives; therefore, they should be regarded as plausible interpretive explanations rather than findings established by the present study. Future research should examine which professional, educational, and organisational characteristics enable some midwives to maintain respectful practices despite demanding working conditions.

Midwives’ repeated emphasis on workload, staffing shortages, institutional routines, and physician-centred decision-making also requires cautious interpretation. These accounts may accurately reflect substantial structural barriers to respectful maternity care; however, they may simultaneously function as professionally protective explanations that shift attention away from individual responsibility. Because obstetric violence is a sensitive and potentially stigmatising concept, participants may have found it easier to attribute problematic practices to the healthcare system than to acknowledge the possible influence of personal attitudes, communication styles, or discretionary behaviours. This interpretation does not imply that organisational constraints were insincere or unimportant. Rather, it suggests that structural explanations may serve both as descriptions of genuine working conditions and as narratives through which professionals manage moral discomfort, professional identity, and accountability. The possibility of social desirability should therefore be considered when interpreting midwives’ accounts.

The Turkish context is also important for interpreting the findings. Türkiye has high levels of medicalised childbirth, and discussions of medicalisation often focus on caesarean section rates. However, this study shows that medicalisation is also relevant to vaginal birth through practices such as induction, episiotomy, continuous monitoring, and routine institutional care processes. Women’s reports of intervention-intensive care, insufficient explanation, and limited involvement in decision-making suggest that vaginal birth may also be experienced as medicalised and disempowering when women are not adequately informed or actively included in care decisions. Therefore, improving childbirth experiences in Türkiye requires attention not only to the mode of birth, but also to communication, consent, privacy, emotional support, and the relational quality of intrapartum care.

The findings should also be interpreted in relation to the gendered sociocultural context of childbirth in Türkiye. Previous research has shown that pregnancy and childbirth are closely intertwined with gender roles and that women’s reproductive experiences may be shaped by patriarchal family and social expectations [28]. Research on the medicalisation of childbirth in Türkiye has further suggested that traditional patriarchal norms and modern medical authority may operate together, encouraging women to defer to professional expertise even when hospital birth is experienced as unpleasant, disempowering, or inconsistent with their preferences [29]. Within such a context, compliance may be interpreted as being a “good” or cooperative patient, whereas questioning procedures or refusing recommendations may be experienced as difficult or socially inappropriate. This may contribute to women’s limited participation in decision-making and to the normalisation of interventions performed with insufficient explanation or meaningful consent. However, these sociocultural mechanisms were not directly examined in the present study and should therefore be considered an interpretive contextual explanation rather than a conclusion derived directly from participants’ accounts. Future research should explicitly explore how gender norms, family expectations, socioeconomic position, and perceptions of professional authority influence women’s ability to express preferences, ask questions, and refuse interventions during childbirth.

A further contextual consideration concerns deference to medical hierarchy. Women’s limited participation in decision-making may be shaped not only by insufficient information but also by expectations that medical authority should not be questioned during childbirth. At the same time, midwives’ accounts indicated that hierarchical relations also operated within the healthcare team, particularly through physician-dominated decision-making and restricted midwifery autonomy. These parallel forms of deference may reinforce one another: women may hesitate to question professionals, while midwives may hesitate to challenge physician-led decisions or established institutional routines. Consequently, practices may become normalised even when women experience them as distressing and midwives recognise that they are not fully consistent with respectful maternity care. However, the present study did not directly assess attitudes towards authority or compare hierarchical cultures across countries. It therefore cannot establish that obedience to medical hierarchy is stronger in Türkiye than elsewhere. Rather, the findings suggest that professional and institutional hierarchy is a relevant contextual mechanism that should be examined explicitly in future comparative research.

The findings have several implications for clinical practice and policy in Türkiye. First, maternity units could implement a brief intrapartum communication and consent protocol requiring healthcare professionals to explain the purpose, expected benefits, possible discomforts, and available alternatives before non-emergency examinations and interventions. Rather than treating consent as a one-time signature, this protocol should support ongoing verbal confirmation and documentation of women’s preferences throughout labour and birth. Visual consent prompts or bedside checklists could be used to facilitate implementation during busy shifts. Second, hospitals could introduce structured respectful maternity care rounds in which a designated midwife periodically assesses whether each woman has received adequate information, emotional support, privacy, opportunities for mobility, and involvement in decision-making. A confidential post-birth feedback mechanism, such as a short digital questionnaire accessed through a quick response (QR) code, could allow women to report respectful and disrespectful care without discussing these concerns directly with the clinical team responsible for their care. Aggregated feedback could then be reviewed regularly by maternity-unit quality committees. Third, simulation-based interprofessional training should involve midwives, obstetricians, nurses, residents, and support staff rather than focusing solely on individual midwives. Training scenarios adapted to common hospital situations in Türkiye could address informed consent under time pressure, refusal of routine procedures, privacy in crowded birth environments, communication with distressed women, culturally sensitive care, and respectful management of disagreement between women and professionals. Including women’s anonymised narratives in these programmes may help professionals recognise how routine practices are experienced by care recipients. Fourth, maternity units could pilot a midwife-led respectful care advocate role. During each shift, an experienced midwife could be assigned responsibility for supporting women’s preferences, facilitating communication between women and physicians, identifying privacy or consent concerns, and helping staff resolve ethical tensions arising from institutional routines. This role may be particularly relevant in physician-centred settings where individual midwives have limited authority to challenge established decisions. Finally, system-level improvements should include staffing standards based on the number and acuity of labouring women, protected time for communication and emotional support, restricted access to birth rooms, and institutional review of routine practices such as induction, continuous monitoring, enema, fasting, mobility restriction, and episiotomy. These interventions should be evaluated through implementation studies examining women’s experiences, professional behaviour, organisational feasibility, and sustainability across public and university hospitals in Türkiye.

Taken together, the findings indicate that obstetric violence cannot be adequately explained by either individual misconduct or organisational constraints alone. Women’s accounts demonstrate how routine practices may be experienced as traumatic when communication, consent, dignity, and emotional support are insufficient, whereas midwives’ accounts reveal the professional hierarchies and institutional conditions within which these practices are enacted. At the same time, structural explanations should not remove professional accountability, because individual communication, ethical sensitivity, and discretionary conduct may either intensify or mitigate the effects of demanding working conditions. Preventing obstetric violence therefore requires a dual approach: institutional reforms that reduce structural barriers and strengthen midwives’ autonomy, together with professional accountability mechanisms that ensure respectful communication, meaningful consent, privacy, and woman-centred decision-making in everyday practice.

### Strengths and Limitations

This study has several strengths. First, it explored obstetric violence from the perspectives of both women and midwives, enabling a more comprehensive understanding of how the same maternity care processes may be experienced by women and interpreted by care providers. Second, the qualitative design enabled an in-depth exploration of lived experiences, meanings, and perceptions related to respectful maternity care, traumatic childbirth, and systemic constraints in hospital birth settings. Third, the inclusion of participants associated with one university hospital and two public hospitals provided contextual variation and enriched the interpretation of the findings. The use of direct quotations, independent coding, investigator discussions, and systematic data management further supported the credibility and transparency of the analytical process.

Several limitations should nevertheless be considered. The study was conducted within a limited number of hospital settings in Türkiye, which may restrict the transferability of the findings to maternity care settings with different organisational, cultural, or clinical characteristics. Different non-probability sampling strategies were used for the two participant groups: women were recruited through snowball sampling, whereas midwives were recruited purposively. Women were also eligible only if they subjectively perceived their childbirth experience as negative or traumatic. The findings therefore reflect the accounts of a specific group of women and should not be interpreted as representing the experiences of all women who give birth in hospital settings. This selection approach may also have resulted in greater representation of negative or distressing childbirth experiences.

Women’s accounts were based on retrospective self-report within the first year after birth. Although retrospective narratives were appropriate for exploring the meanings attributed to traumatic childbirth, recall bias may have influenced how events were remembered, reconstructed, and narrated over time. Emotional responses following childbirth may also have shaped the salience and interpretation of particular experiences.

Midwives’ accounts may have been influenced by social desirability and professional role expectations, particularly because the interviews addressed sensitive issues involving communication, consent, institutional routines, and obstetric violence. Although confidentiality, individual interviewing, neutral questioning, and the absence of right or wrong answers were emphasised, these strategies could reduce but not eliminate this bias. Some midwives may have underreported practices perceived as professionally undesirable or may have emphasised workload and organisational constraints in ways that reduced the perceived role of individual attitudes and professional responsibility.

The study did not include direct clinical observation of childbirth care. Consequently, reports concerning information provision, informed consent, privacy, induction, episiotomy, emotional support, and professional interactions represent participants’ perceptions and interpretations rather than independently verified accounts of clinical practice. This does not diminish the value of the findings for understanding lived experience; however, it limits conclusions about the objective frequency, circumstances, or intent of the practices described. The findings should therefore be interpreted as experiential and professional accounts rather than as observational evidence of specific clinical events.

Obstetricians, hospital administrators, and other healthcare professionals were not included in the study. Their exclusion limited the examination of physician-led decision-making, institutional policies, professional hierarchies, and organisational responsibility from the perspectives of other actors involved in maternity care. Including these groups in future studies could provide a more comprehensive understanding of how individual, professional, and institutional factors interact.

Although the interview guides underwent multidisciplinary review by five academic experts, a separate pilot interview was not conducted. Therefore, the practical functioning of the guides and participants’ interpretations of the questions were not formally tested with individuals resembling the study participants before data collection. Furthermore, women were interviewed online, whereas midwives were interviewed face-to-face. These different interview formats may have influenced rapport, disclosure, and the observation of non-verbal communication.

The inclusion of women who gave birth between 34 and 41 gestational weeks should also be considered. Late preterm birth may influence women’s emotional responses and perceptions of childbirth as traumatic, but this factor was not analysed separately.

## 5. Conclusions

This study showed that women experienced obstetric violence primarily through emotional distress, loss of autonomy, inadequate information, compromised consent, privacy violations, and unmet emotional needs during childbirth. Midwives recognised the importance of respectful and woman-centred care but frequently interpreted deficiencies in care in relation to workload, institutional routines, physician-centred decision-making, restricted professional autonomy, and organisational constraints.

The comparison of the two perspectives indicates that obstetric violence should not be understood solely as either individual misconduct or a consequence of structural conditions. Rather, it emerges through the interaction of institutional arrangements, professional hierarchies, routine clinical practices, and everyday interpersonal conduct. Structural constraints may restrict professionals’ capacity to provide individualised care, but they do not eliminate the importance of communication, ethical sensitivity, discretionary behaviour, and professional accountability.

Prevention therefore requires interventions at both the interpersonal and institutional levels. Maternity care should ensure continuous and meaningful informed consent, clear communication, protection of privacy and dignity, emotional support, and women’s active participation in decision-making. At the organisational level, staffing and workload conditions, interprofessional collaboration, institutional routines, and midwives’ professional autonomy should be improved. These reforms should be accompanied by mechanisms that support reflective practice and accountability and enable healthcare professionals to recognise and challenge normalised practices that women may experience as coercive, disrespectful, or traumatic.

## Figures and Tables

**Table 1:** Characteristics of the women and midwives participating in the study.

**Panel A. Sociodemographic characteristics of participating women (N = 21)**			
**Variable**	Category		
**Age (years)**	Mean ± SD	30.4 ± 7.4	
		**n**	**%**
**Marital status**	Married	19	90.5
	Single	2	9.5
**Family type**	Nuclear family	17	81.0
	Extended family	2	9.5
	Divorced/single-parent family	2	9.5
**Duration of marriage (years)**	Mean ± SD	7.2 ± 3.9	
**Marital satisfaction**	Moderate	4	19.0
	Good	9	42.9
	Very good	8	38.1
**Education level**	Primary education	1	4.8
	High school graduate	7	33.3
	University graduate	13	61.9
**Employment status**	Homemaker	6	28.6
	Civil servant	13	61.9
	Self-employed	1	4.8
	Not reported	1	4.8
**Having previous children before the index birth**	Yes	19	90.5
	No	2	9.5
**Income status**	Income less than expenses	8	38.1
	Income equal to expenses	7	33.3
	Income greater than expenses	6	28.6
**Panel B. Professional characteristics of participating midwives (N = 20)**			
**Age, years**	Mean ± SD	33.5 ± 11.7	
**Total professional experience, years**	Mean ± SD	11.2 ± 11.9	
**Experience in labour and delivery units, years**	Mean ± SD	9.1 ± 8.6	

Note. Data are presented as n (%) or mean ± standard deviation. Duration of marriage was calculated only for married women. For midwives, only age, total professional experience, and experience in labour and delivery units were collected. Participant characteristics are presented in aggregate form because individual-level characteristics were not retained in a format linked to the participant codes used in the qualitative quotations. Aggregate reporting was maintained to avoid retrospective reconstruction of participant-level data and to protect participant confidentiality. Percentages may not total 100 because of rounding.

**Table 2:** Women’s self-reported childbirth-related practices and experiences (N = 21).

Variable/Question	Response	n	%	Mean	SD	Min–Max
Gestational age at birth (weeks)		21		38.24	1.95	34–41
Was labour induction (oxytocin) applied?	Yes	8	38.1	–	–	–
	No	13	61.9	–	–	–
Was enema applied?	Yes	6	28.6	–	–	–
	No	15	71.4	–	–	–
Was informed consent obtained?	Yes	12	57.1	–	–	–
	No	9	42.9	–	–	–
Was information provided during labour?	Yes	15	71.4	–	–	–
	No	6	28.6	–	–	–
Were you exposed to physical violence during childbirth?	Yes	2	9.5	–	–	–
	No	19	90.5	–	–	–
Was episiotomy performed?	Yes	11	52.4	–	–	–
	No	10	47.6	–	–	–
Was skin-to-skin contact provided after birth?	Yes	14	66.7	–	–	–
	No	7	33.3	–	–	–

Note. n = number of participants; SD = standard deviation. Data are based on women’s self-reports regarding their individual childbirth experiences. Percentages were calculated using the total sample size. These descriptive data were used only to provide contextual background for the qualitative findings and were not intended for inferential interpretation.

**Table 3:** Midwives’ reports of routine practices and organisational conditions in delivery rooms (N = 20).

Question	Yes n (%)	No n (%)
Is there a separate labour room for each woman?	12 (60)	8 (40)
Is information provided to women about the process?	14 (70)	6 (30)
Is routine induction applied?	2 (10)	18 (90)
Is routine enema applied?	8 (40)	12 (60)
Is continuous non-stress test (NST) routinely used?	13 (65)	7 (35)

Note. These data reflect midwives’ self-reports regarding perceived routine practices and organisational conditions in their working settings; they do not represent direct clinical observation. Because women’s reports reflect individual childbirth experiences and midwives’ reports reflect perceived routine practices, the descriptive findings in Table 2 and Table 3 are not intended for direct statistical comparison.

**Table 4:** Themes, subthemes, illustrative quotations, and comparative interpretation of women’s and midwives’ perspectives.

Main Theme	Subtheme	Women’s Illustrative Quotation	Midwives’ Illustrative Quotation	Comparative Interpretation
**Lack of Respectful Maternity Care**	Inadequate information and compromised informed consent	*“No, I was not given enough information, and my consent was not obtained. It was more like they told me and then did it. There were times when I did not receive answers to all my questions.”* (P5)	*“Women should be informed before every procedure, but during busy shifts this is not always possible.”* (M5)	Women interpreted inadequate information and limited informed consent as a loss of autonomy and exclusion from decision-making. Midwives acknowledged the importance of informed consent but associated deficiencies with time constraints, workload, and routine clinical practice.
	Interventions perceived as imposed	*“When I could not push correctly, they applied pressure to my abdomen. I know they did it for my baby, but if they had explained how painful it would be, I could have prepared myself.”* (P3)	*“Sometimes interventions are initiated before the woman has fully understood what is happening.”* (M8)	Women experienced interventions as physically and emotionally distressing when they were insufficiently explained. Midwives recognised that communication surrounding interventions was sometimes inadequate, particularly within physician-led clinical routines.
	Compromised privacy and dignity	*“There were so many people around me while I was giving birth that I felt as though I were an object on display.”* (P8)	*“Compared with my first years in practice, privacy has improved, but too many people still enter and leave the room. Physicians, midwives, nurses and support staff come in repeatedly, doors are often left open, and women become very uncomfortable.”* (M8)	Women described privacy violations as threats to dignity, bodily integrity, and emotional comfort. Midwives similarly recognised privacy as a persistent challenge, attributing these breaches to organisational routines and the physical organisation of maternity units.
**Women’s Emotional Needs During Childbirth**	Emotional vulnerability during childbirth	*“I felt completely alone. I had never felt so lonely in my life.”* (P19)	*“We try to support women emotionally, but sometimes we are responsible for too many women at the same time.”* (M10)	Women experienced fear, loneliness, helplessness, and emotional abandonment during childbirth. Midwives acknowledged these emotional needs but reported that staffing shortages and workload limited their ability to provide continuous emotional support.
	Need for emotional reassurance	*“Even a few encouraging words would have made a difference.”* (P7)	*“Women need reassurance throughout labour, but maintaining continuous communication is difficult during busy shifts.”* (M13)	Women regarded reassurance and compassionate communication as essential components of a positive childbirth experience. Midwives shared this perspective but described organisational pressures as limiting opportunities for sustained supportive communication.
	Communication failures amplifying traumatic experiences	*“I was afraid because I did not know what was happening. No one explained anything, and that made everything much worse.”* (P11)	*“We try to explain every procedure, but when several women are in labour simultaneously, communication inevitably becomes shorter.”* (M16)	Women linked poor communication directly to increased fear, uncertainty, and traumatic perceptions of childbirth. Midwives associated communication gaps primarily with workflow pressures rather than lack of awareness or professional commitment.
**Systemic Constraints Affecting Midwifery Practice**	Restricted professional autonomy	*“I felt like everything was happening around me, but I was not part of the decisions.”* (P5)	*“Sometimes we know what would be best for the woman, but we cannot act independently.”* (M7)	Women perceived exclusion from decision-making as a loss of control over childbirth, whereas midwives described limited professional autonomy as a barrier to providing evidence-based, woman-centred care.
	Physician-dominated decision-making	*“Everything felt rushed, as if I were part of a routine process rather than an individual.”* (P3)	*“Most decisions are made by physicians, and our influence on clinical management is limited.”* (M16)	Women experienced standardised, physician-directed care as impersonal and procedure-oriented. Midwives identified hierarchical decision-making as a structural mechanism contributing to these experiences.
	Moral distress in midwifery practice	*“I wanted someone to understand what I was feeling, not just complete the procedures.”* (P8)	*“We often feel caught between what we believe is best for women and what the system allows us to do.”* (M19)	Women experienced the consequences of organisational constraints as loss of personalised care and emotional neglect. Midwives described these same constraints as generating ethical tension and moral distress when professional values conflicted with institutional expectations. Together, these findings suggest that obstetric violence emerges through the interaction of interpersonal care practices and structural conditions within maternity care systems.

Note. This table presents the three main themes, related subthemes, illustrative quotations, and comparative interpretation of women’s and midwives’ perspectives. Women were coded as P1–P21 and midwives as M1–M20. The dash indicates that no direct quotation was selected for that subtheme in the current analysis. The table is intended to complement the qualitative findings and does not represent a quantitative comparison.

## Data Availability

The data generated and analysed during the current study are not publicly available due to the sensitive nature of the interviews and the need to protect participant confidentiality. Anonymised excerpts supporting the findings are included in the article. Additional anonymised data may be made available from the corresponding author upon reasonable request, subject to ethical approval and confidentiality restrictions.

## Supplementary Materials

The following supporting information can be downloaded at: https://www.mdpi.com/article/10.3390/healthcare14162579/s1, Supplementary File S1: Semi-Structured Interview Guide for Women with Traumatic Childbirth Experiences and Midwives.
