# Supplementary material for: Obstetric Violence Through the Perspectives of Women and Midwives in Hospital Birth Settings: A Qualitative Study
Source: Healthcare (Basel). 2026 Aug 17;14(16):2579. doi: 10.3390/healthcare14162579 (PMC13512219; doi:10.3390/healthcare14162579)
Supplement: Supplementary file 1 [file healthcare-14-02579-s001.zip › healthcare-4413169-supplementary.pdf]

Supplementary File S1. Semi-Structured Interview Guide for Women with Traumatic Childbirth Experiences and Midwives

Part A. Interview Guide for Women with Traumatic Childbirth Experiences

Opening Questions

1. Could you tell me about your childbirth experience?
   Probe: How did you feel during labour and birth?
2. What were your expectations before childbirth, and how did your actual experience compare with those expectations?

Experiences During Labour and Birth

3. How would you describe the care you received from healthcare professionals during labour and birth?
   Probe: Could you give an example?
4. Can you describe how information about examinations, procedures, or interventions was communicated to you during labour and birth?
   Probes: What information was provided? At what point was it provided? How understandable and sufficient did you find it?
5. How were decisions made regarding examinations, procedures, or interventions during your labour and birth?
   Probes: How were you involved in these decisions? How were your agreement, refusal, or preferences addressed? How did this process make you feel?
6. Can you describe how your opinions, preferences, or birth-related wishes were addressed during labour and birth?
   Probe: Can you provide an example of a situation in which your preferences were considered or not considered?

Communication and Emotional Support

7. How would you describe your communication with the healthcare professionals?
8. Can you describe the emotional support you received, or did not receive, during labour and birth?
   Probes: Who provided support? What behaviours or interactions were helpful or unhelpful?
9. Can you describe any behaviours or attitudes that made you feel respected, safe, or comfortable?
10. Can you describe any situations or interactions that made you feel ignored, frightened, or distressed?

Privacy and Birth Environment

11. How would you describe the birth environment?
12. Can you describe how your privacy, bodily boundaries, and dignity were managed during labour and birth?
    Probes: Were there any situations in which you felt comfortable, exposed, respected, or uncomfortable?

Reflection

13. Looking back, what aspects of your childbirth experience would you like to see improved?
14. Is there anything else you would like to share about your childbirth experience?

Part B. Interview Guide for Midwives

Professional Background

1. Could you briefly describe your professional experience in labour and delivery care?
2. How would you describe the birth environment in your institution?

Routine Maternity Care Practices

3. Could you describe the routine care practices commonly performed during labour and birth?
4. How are women informed about examinations and interventions?
5. How is informed consent obtained before procedures?
6. How are women's privacy and dignity protected during labour and birth?
7. How are women's emotional needs addressed during labour?

Respectful Maternity Care

8. What does respectful maternity care mean to you?
9. How is respectful maternity care implemented in your daily practice?

Obstetric Violence

10. What does the term obstetric violence mean to you?
11. Can you describe any practices you have observed that might be considered obstetric violence? Probe: Could you describe some examples?
12. Which routine practices do women most frequently express discomfort or dissatisfaction with?

Organisational Factors

13. What organisational or systemic factors influence the quality of maternity care in your institution?
14. What barriers make it difficult to provide respectful maternity care?

Recommendations

15. In your opinion, what changes are needed to reduce obstetric violence and improve respectful maternity care?
16. Is there anything else you would like to add?

Note. Follow-up probes were used flexibly when clarification, elaboration, or concrete examples were needed.
